# Inflammatory cytokines can be monitored in exhaled breath particles following segmental and inhalation endotoxin challenge in healthy volunteers

**DOI:** 10.1038/s41598-022-09399-z

**Published:** 2022-04-04

**Authors:** Olaf Holz, Meike Müller, Saskia Carstensen, Anna-Carin Olin, Jens M. Hohlfeld

**Affiliations:** 1 Department of Clinical Airway Research, Fraunhofer Institute for Toxicology and Experimental Medicine ITEM, 30625 Hannover, Germany; 2 German Center for Lung Research (BREATH), Hannover, Germany; 3 Occupational and Environmental Medicine, School of Public Health and Community Medicine, Institute of Medicine, Gothenburg University, Gothenburg, Sweden; 4 Department of Respiratory Medicine, Hannover Medical School (MHH), Hannover, Germany

**Keywords:** Biomarkers, Biomarkers, Medical research

## Abstract

Particles in exhaled air (PEx) are generated when collapsed small airways reopen during breathing. PEx can be noninvasively collected by particle impaction, allowing the analysis of undiluted epithelial lining fluid (ELF). We used the endotoxin (LPS) challenge model to proof the concept that PEx can be used to monitor inflammatory changes in the lung. In this pilot study PEx were collected from ten healthy nonsmoking subjects using the PExA^®^ instrument twice before and twice after a segmental LPS challenge (5, 21 h). Following a 4-week washout period, PEx were collected during the week before and 5 h after a whole lung LPS inhalation challenge. PEx biomarkers were compared to blood, bronchoalveolar lavage (BAL) following segmental challenge and induced sputum (ISP) following inhalation challenge. A clear LPS-induced inflammatory response was detectable in BAL fluid, ISP and blood. Albumin and surfactant–protein D were detectable in all PEx samples. While most baseline cytokines were close to or below the detection limit, the median (IQR) IL-6 and IL-8 concentrations in PEx increased significantly after segmental (0.04 (0.03; 0.06) fg/ng PEx; 0.10 (0.08; 0.17) fg/ng PEx) and inhalation LPS challenge (0.19 (0.15; 0.23) fg/ng PEx; 0.32 (0.23; 0.42) fg/ng PEx). Using a highly sensitive analysis platform, we were able to detect a cytokine response in PEx during the early phase of LPS-induced inflammation. This will broaden the spectrum of applications for this noninvasive method to monitor inflammatory processes in the lung, including its use in clinical trials for respiratory drug development.

Trial registration: The study has been registered on 07.02.2017 at Clinicaltrials.gov (NCT03044327).

## Introduction

Noninvasive monitoring of airway inflammation as a surrogate for disease activity is important both in basic respiratory research and in clinical airway research, e.g., to assess the efficacy of novel drugs. In this study, we used the endotoxin (LPS) challenge model^1–3^ to test the performance of exhaled breath particle analysis, a novel noninvasive tool to collect undiluted epithelial lining fluid from the peripheral airways^4–6^.

It is widely accepted that reopening of collapsed small airways during normal breathing is the dominant mechanism for PEx generation in the peripheral lung^4,6,7^. PEx can be collected by exhalation through cooled sampling systems as exhaled breath condensate or undiluted by impaction using, e.g., the PExA device^6^. The latter includes a particle counter to determine the number and size spectrum of the exhaled particles^8^. It is of utmost importance to compute the number and mass of PEx that are actually collected because despite using standardized breathing maneuvers, particle emission shows very large interindividual differences^9^, which could also contribute to increased variability in cross-sectional EBC studies and the insufficient reproducibility discussed in the last ERS Taskforce report^10^. We therefore used BAL, ISP and serum as reference for the PEx analysis.

LPS challenge of single lung lobes by bronchoscopic instillation or of the whole lung by controlled inhalation are well-established methods to induce a transient and well-controlled inflammatory response in the lungs of healthy subjects. It has a clear advantage for assessing the clinical value of novel inflammation-monitoring tools compared to using patients with chronic airway inflammation because it is not affected by potentially varying states of inflammation in patients. Broad experience has been gained in using this challenge model as an experimental research tool and for profiling the pharmacodynamic efficacy of investigational new drugs in early stages of clinical development^1,2^.

In this study, we included ten healthy volunteer subjects who underwent both segmental and inhalation challenges in two experimental blocks separated by a 4-week washout period. The inflammatory response was assessed by BAL after segmental challenge^2^ and by induced sputum after inhalation challenge^3^. Blood samples were also collected at the respective time points. This complex study was designed to further characterize the LPS challenge model for future applications and to test novel noninvasive tools to monitor airway inflammation. These included specific gas-enhanced MRI^11^, the analysis of volatile organic compounds^12^ and specific cell analysis by flow cytometry and Chipcytometry^13^. Here, we present proof-of-concept data for monitoring cytokines in PEx following LPS challenge.

## Methods

### Study design

**Figure 1:**
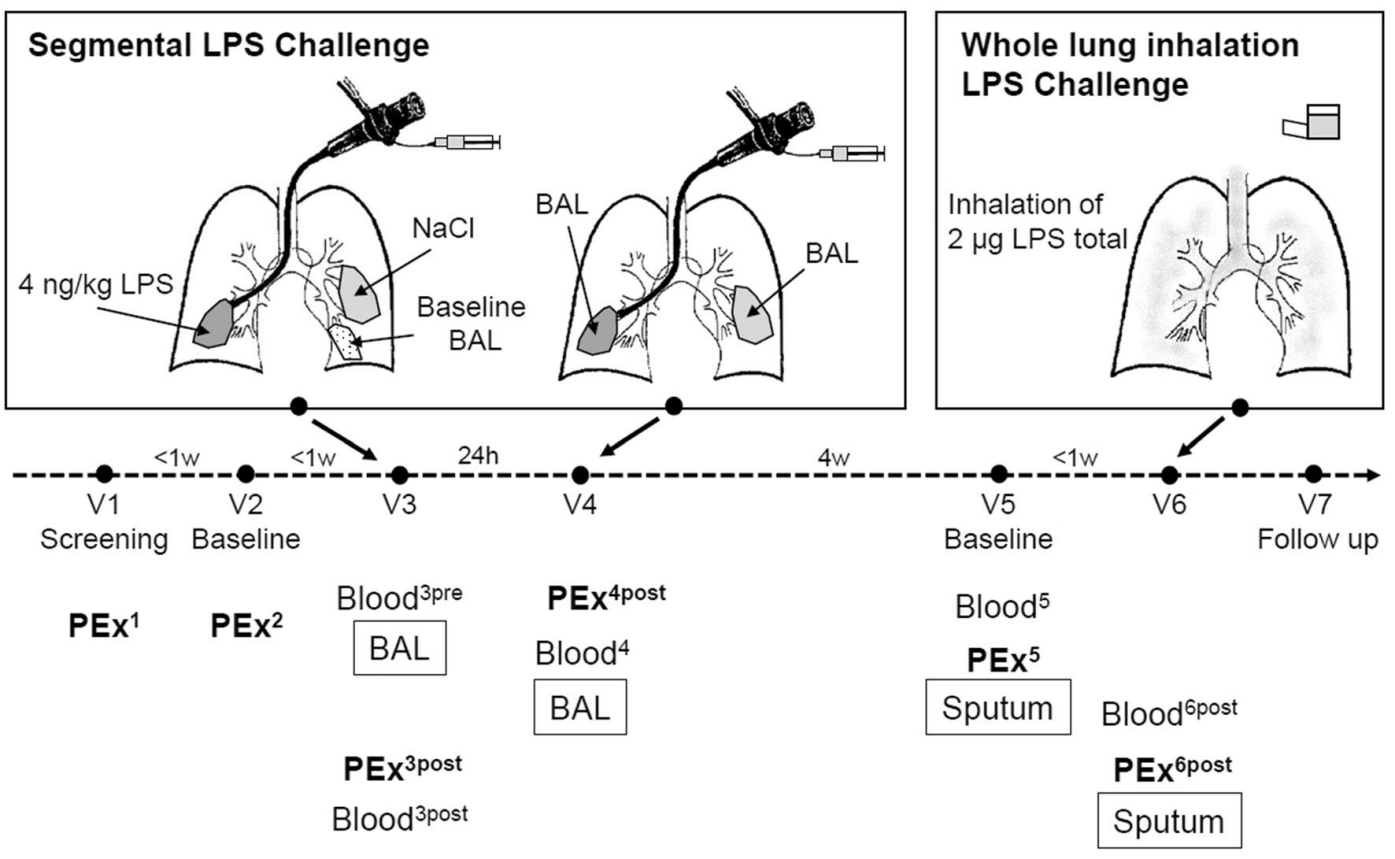
Study design. The study was designed to test the ability of novel noninvasive methods to detect airway inflammation. Here we focus on exhaled breath particles (PEx). *BAL* bronchoalveolar lavage, *V* visit (index pre: refers to samples taken before a challenge, post: refers to samples taken after a challenge), *NaCl* isotonic saline solution, *LPS* endotoxin, *w* week, *h* hours. PEx samples were collected on V3 in the afternoon 5 h after the segmental challenge. On V4 PEx were sampled prior to the BAL procedure 21 h after the LPS challenge. On visit 5 and 6 PEx were collected before the sputum induction. On V6 this was done 5 h following the inhalation LPS challenge.

The study is outlined in Fig. 1. Volunteers underwent a segmental LPS challenge and, following a four-week washout period, a whole lung inhalation challenge. Exhaled breath particles (PEx) were sampled during a screening visit (V1), a first baseline visit (V2), 5 h following the segmental LPS challenge (V3), 21 h after segmental LPS challenge (V4), at a second baseline visit (V5) and 5 h following the inhalation LPS challenge (V6). Blood samples were collected in the morning of V3 before the segmental LPS challenge, 6 h and 21 h after segmental challenge, and on V5 and 6 h after inhalation challenge on V6.

The segmental and inhalation challenges were performed as previously reported^1–3,13^. The procedures of bronchoalveolar lavage and sputum induction have also recently been published^13^ and are described in detail in Additional file 1.

### Subjects

Ten healthy, nonsmoking subjects (7 male and 3 females, smoking history < 1 pack-year, mean (SD) age 38 ± 10 years) with normal lung function (FEV_1_: 101 ± 14% pred.) were included. The study was conducted in accordance with local laws, regulations and GCP guidelines (CPMP/ICH/135/95), and the protocol was approved by the ethics committee of the Hannover Medical School and registered on 07.02.2017 at Clinicaltrials.gov (NCT03044327). Every subject provided written informed consent after being fully informed about the study.

### PEx sampling

Breath samples were collected using a commercially available device for particle sampling (Particles in Exhaled Air [PExA], Gothenburg, Sweden. For a schematic graph of the device please refer to^14^). We aimed to sample 240 ng at each collection but limited the collection time to a maximum time of 30 min (Table S1, see Additional file 1). Estimations of the PEx mass are based on the data of the optical particle counter (Grimm Aerosol, Ainring, Germany), which is included in the PExA instrument. The number of PEx in 8 size ranges between 0.4 and 4.6 μm were determined. With this information and assuming spherical particles with a unit density of 1000 kg m^−3^, the mass of the collected particles can be estimated^4^. Participants were asked to perform repetitive breathing maneuvers, as described by Bake et al.^4^, to increase PEx emissions. PEx were collected on Teflon membranes (0.45 µm PTFE Membranes 25 mm, Merck FHLC02500, Darmstadt, Germany).

### PEx analysis

Half of each collection filter was extracted using an adapted method based on a recommended procedure by PExA. In short, 200 µL of RIPA buffer, including a protease inhibitor cocktail (Merck, Darmstadt, Germany), was added to the membrane in a 1.7 mL tube and incubated at 30 °C for 30 min under constant rapid agitation. The fluid was transferred to an empty 1.5 mL low binding tube (Eppendorf, Hamburg, Germany) and directly used for analysis.

The Meso Scale Discovery (MSD) platform was used for analysis: S-Plex Human IL-6 Kit with a lower limit of detection (LLOD) of 1.1 fg/mL, V-Plex Human IL-8 with an LLOD of 0.04 pg/mL, combined R-Plex for MPO and PSP-D (SP-D) with LLODs of 11 pg/mL and 37 pg/mL, R-Plex Human albumin with an LLOD of 155 pg/mL.

### Data analysis

Due to the exploratory character of this pilot study, a power calculation was not performed. Data are presented as median and IQR. Nonnormally distributed data were log-transformed prior to analysis. Parametric methods were used for comparison. For potential LPS effects, we included the presegmental challenge and the two postsegmental challenge time points in the ANOVA. The pre- and postinhalation time points were analyzed separately. Significance level was set to 0.05.

### Ethics approval and consent to participate

The study was conducted in accordance with local laws, regulations and GCP guidelines (CPMP/ICH/135/95), and the protocol was approved by the ethics committee of the Hannover Medical School (Approval No. 7193). Every subject provided written informed consent after being fully informed about the study.

## Results

### LPS induced inflammatory response

Tables 1 and 2 summarizes the LPS-induced inflammatory response in BAL fluid, induced sputum, and blood. The segmental LPS challenge induced a massive influx of neutrophils and monocytes into the challenged segment. Tables 1 and 2 shows the cellular data obtained by standard microscopy. A more detailed analysis including flow cytometry and Chipcytometry has recently been published^13^. LPS also caused an increase in the total cell count as well as in the BAL fluid concentration of albumin, IL-6, IL8, and MPO of each subject. The whole lung LPS challenge resulted in a comparable inflammatory response with an increase in the percentage of neutrophils and monocytes and the concentrations of IL-6, IL8 and MPO in the sputum of all subjects. A pronounced inflammatory response was also observed in blood both 6 and 21 h after the segmental challenge and 6 h after the whole lung challenge.

**Table 1:** Summary of the inflammatory response in bronchoalveolar lavage (BAL), blood/serum and PEx for segmental LPS challenge (V1–V4).

Visit			V1	V2	V3pre	V3post	V4	
					Segmental challenge			
					Baseline		Saline	LPS
**BAL**	Macrophages	%			87.8 (84.7; 90.2)		85.8 (77.6; 91.0)	12.4 (8.2; 15.6)***
	Neutrophils	%			1.1 (0.8; 2.4)		3.0 (1.4; 5.6)	64.0 (60.0; 67.3)***
	Lymphocytes	%			7.7 (4.8; 9.0)		6.3 (4.9; 7.1)	2.3 (1.7; 3.4)***
	Monocytes	%			1.7 (0.9; 1.9)		2.0 (1.4; 3.1)	22.6 (18.3; 26.3)***
	Eosinophils	%			0.0 (0.0; 0.1)		0.1 (0.0; 0.2)	0.1 (0.0; 0.6)
	Col. epithelial cells	%			1.8 (1.4; 2.2)		1.4 (0.9; 1.6)	0.1 (0.0; 0.1)***
	Sq. epithelial cells	%			0.1 (0.0; 0.3)		0.0 (0.0; 0.1)	0.0 (0.0; 0.0)
	Macrophages	10^4^/mL			5.8 (5.5; 9.6)		7.7 (5.6; 10.5)	8.6 (6.2; 11.1)
	Neutrophils	10^4^/mL			0.1 (0.1; 0.2)		0.2 (0.1; 0.6)	43.8 (33.2; 67.9)***
	Lymphocytes	10^4^/mL			0.5 (0.4; 0.6)		0.5 (0.4; 0.9)	1.3 (1.1; 2.6)*
	Monocytes	10^4^/mL			0.1 (0.1; 0.2)		0.2 (0.1; 0.3)	13.9 (8.7; 28.6)***
	TCC	10^4^/mL			6.6 (6.2; 11.7)		8.5 (7.0; 11.7)	68.2 (56.0; 99.7)***
	IL6	pg/mL			0.6 (0.4; 0.9)		1.6 (0.9; 2.2)	61.1 (39.9; 71.1)***
	IL8	pg/mL			12.2 (10.2; 15.8)		16.8 (7.6; 22.3)	52.2 (43.9; 65.9)***
	ALB	µg/mL			36.9 (31.2; 39.7)		33.6 (29.4; 41.9)	143.5 (115.8; 185.7)***
	MPO	ng/mL			5.2 (2.9; 9.1)		12.3 (4.8; 20.1)	100.5 (87.8; 141.2)***
	SPD	ng/mL			217.6 (181.2; 242.5)		211.3 (182.9; 256.8)	175.4 (156.6; 202.6)
**Blood**	Neutrophils	%			56.5 (53.3; 60.1)	78.7 (73.8; 82.5)**	64.7 (61.1; 70.8)**	
	Lymphocytes	%			32.1 (29.3; 34.4)	13.1 (12.0; 18.2)**	27.8 (20.8; 29.2)*	
	Monocytes	%			8.8 (6.9; 9.6)	6.8 (5.4; 7.2)*	6.4 (4.9; 7.6)***	
	Eosinophils	%			1.6 (1.0; 2.7)	0.9 (0.4; 1.3)**	1.0 (0.6; 1.7)*	
	Basophils	%			0.7 (0.5; 1.2)	0.4 (0.3; 0.6)**	0.6 (0.4; 0.6)*	
	Leukocytes	G/L			5.0 (4.2; 6.0)	10.6 (9.8; 12.3)***	7.6 (6.3; 8.4)***	
	Erythrocytes	T/L			4.8 (4.5; 5.2)	4.7 (4.6; 4.9)	4.8 (4.7; 5.1)	
	Hemoglobin	g/dL			14.5 (13.3; 14.8)	14.2 (13.8; 14.5)	14.3 (14.1; 14.9)	
	Hematokrit	L/L			0.4 (0.4; 0.4)	0.4 (0.4; 0.4)	0.4 (0.4; 0.4)	
	MCH	pg/Ery			29.3 (28.9; 30.5)	30.1 (29.0; 30.6)	29.4 (29.0; 30.3)	
	MCHC	g/dL			33.9 (33.7; 34.5)	34.2 (33.7; 35.3)	34.6 (34.0; 34.9)	
	MCV	fL			86.9 (84.3; 88.5)	87.1 (85.1; 88.6)	86.7 (83.2; 88.3)	
	Thrombocytes	G/L			222.0 (206.3; 232.3)	220.5 (206.3; 249.0)	218.5 (194.5; 229.0)	
**Serum**	IL6	pg/mL			0.6 (0.5; 0.8)	11.5 (6.0; 20.8)***	2.0 (1.5; 5.2)***	
	IL8	pg/mL			7.4 (4.2; 7.8)	7.6 (4.6; 10.1)	4.0 (3.1; 5.2)*	
	ALB	mg/mL			71.5 (60.6; 77.9)	75.8 (65.0; 80.9)	75.0 (70.9; 77.8)	
	MPO	ng/mL			12.8 (8.2; 16.4)	37.5 (24.3; 67.2)**	38.0 (24.4; 60.5)***	
	SPD	ng/mL			5.2 (3.6; 6.7)	5.8 (4.0; 6.0)	5.7 (3.9; 7.6)	
**PEx**	No. Pex sampled	n	19.0 (15.5; 25.8)	13.0 (9.3; 21.5)		10.5 (8.3; 12.8)*	15.0 (12.5; 22.8)	
	Vol breath sampled	L	86.7 (68.3; 94.7)	56.9 (42.4; 89.9)		39.7 (36.2; 56.9)**	75.8 (57.8; 96.6)	
	Mass sampled	ng	242.7 (157.6; 243.9)	240.3 (228.2; 246.1)		245.1 (240.4; 255.7)	241.5 (238.6; 242.3)	
	IL6	fg/mL	0.0 (0.0; 0.0)	0.0 (0.0; 0.0)		24.3 (14.7; 34.2)***	0.3 (0.1; 1.2)*	
	IL8	pg/mL	0.04 (0.01; 0.04)	0.02 (0.01; 0.04)		0.06 (0.05; 0.10)**	0.04 (0.02; 0.07)	
	ALB	ng/mL	29.8 (27.9; 35.4)	31.3 (25.7; 34.1)		23.9 (18.1; 25.3)	31.3 (19.3; 34.5)	
	MPO	pg/mL	0.1 (0.1; 0.1)	0.1 (0.1; 0.1)		0.1 (0.1; 0.2)	0.3 (0.1; 1.9)	
	SPD	pg/mL	38.4 (28.7; 48.6)	34.1 (18.2; 37.9)		22.9 (17.3; 38.9)	22.5 (14.6; 31.0)	

PEx: concentration in 200 µL eluate from half a filter membrane (PEx mean mass 120 ng). Concentration*0.2/120 ng will give the estimated concentration in ELF.

*p < 0.05,**p < 0.01,***p < 0.001. Median and IQR.

**Table 2:** Summary of the inflammatory response in induced sputum, blood/serum and PEx for inhalation LPS challenge (V5–V6).

Visit			V5	V6post
			Inhalation challenge	
			Baseline	LPS
**ISP**	Macrophages	%NS	54.5 (47.3; 63.6)	6.4 (5.1; 12.0)***
	Neutrophils	%NS	32.0 (18.4; 41.1)	77.9 (70.7; 86.3)***
	Lymphocytes	%NS	1.8 (1.2; 4.2)	2.4 (1.8; 4.8)
	Monocytes	%NS	1.6 (1.3; 2.0)	5.6 (4.0; 7.7)*
	Eosinophils	%NS	0.0 (0.0; 0.1)	0.1 (0.1; 0.5)
	Col. epithelial cells	%NS	4.0 (2.9; 7.0)	0.4 (0.0; 2.0)
	Sq. epithelial cells	%	3.4 (2.5; 7.8)	1.0 (0.6; 2.3)*
	Macrophages	10^4^/mL	2.2 (1.4; 3.1)	1.7 (1.2; 2.1)
	Neutrophils	10^4^/mL	1.9 (1.2; 3.1)	13.6 (5.4; 22.7)*
	Lymphocytes	10^4^/mL	0.1 (0.1; 0.2)	0.4 (0.4; 0.5)**
	Monocytes	10^4^/mL	0.1 (0.1; 0.1)	0.9 (0.4; 1.3)***
	TCC	10^4^/mL	5.2 (3.7; 8.6)	17.7 (8.5; 25.9)**
	IL6	pg/mL	11.7 (10.0; 28.8)	98.3 (58.9; 128.1)***
	IL8	ng/mL	13.5 (5.8; 15.7)	25.2 (16.8; 34.9)***
	ALB	µg/mL	520 (345; 580)	566 (465; 723)*
	MPO	ng/mL	4194 (2275; 5273)	12550 (7675; 20985)***
	SPD	ng/mL	52.5 (21.7; 67.4)	88.4 (28.8; 125.1)
**Blood**	Neutrophils	%	54.8 (51.3; 58.4)	78.0 (74.4; 81.6)***
	Lymphocytes	%	33.7 (29.5; 35.1)	13.8 (11.3; 17.5)***
	Monocytes	%	9.1 (7.2; 9.8)	6.3 (5.2; 6.8)***
	Eosinophils	%	3.3 (1.3; 3.8)	0.9 (0.6; 1.5)***
	Basophils	%	0.8 (0.6; 1.1)	0.4 (0.3; 0.5)**
	Leukocytes	G/L	4.7 (3.9; 5.7)	11.0 (10.7; 11.7)***
	Erythrocytes	T/L	4.8 (4.4; 5.1)	4.8 (4.3; 4.9)*
	Hemoglobin	g/dL	14.0 (13.1; 14.7)	14.0 (13.3; 14.6)
	Hematokrit	L/L	0.4 (0.4; 0.4)	0.4 (0.4; 0.4)
	MCH	pg/Ery	29.5 (28.9; 30.3)	29.7 (29.2; 30.5)
	MCHC	g/dL	33.3 (33.2; 34.6)	34.4 (34.2; 34.8)
	MCV	fL	88.7 (85.5; 89.5)	86.7 (83.4; 89.8)
	Thromboytes	G/L	222.0 (214.0; 255.0)	225.5 (216.5; 244.8)
**Serum**	IL6	pg/mL	0.6 (0.5; 0.7)	5.6 (4.5; 7.8)***
	IL8	pg/mL	5.1 (4.5; 7.3)	8.6 (6.0; 12.4)**
	ALB	mg/mL	65.2 (58.2; 78.6)	72.3 (60.9; 79.6)
	MPO	ng/mL	20.2 (9.4; 38.3)	26.3 (20.9; 54.0)*
	SPD	ng/mL	4.4 (3.7; 6.9)	5.9 (3.6; 7.5)
**PEx**	No. Pex sampled	n	16.5 (12.3; 20.0)	17.0 (12.8; 21.0)
	Vol breath sampled	L	61.9 (53.5; 80.4)	64.4 (54.7; 89.8)
	Mass sampled	ng	248.1 (240.9; 250.6)	242.1 (230.9; 244.9)
	IL6	fg/mL	0.0 (0.0; 0.0)	114.7 (88.7; 137.4)***
	IL8	pg/mL	0.03 (0.03; 0.04)	0.19 (0.14; 0.25)***
	ALB	ng/mL	27.7 (24.8; 29.6)	36.2 (30.3; 38.0)**
	MPO	pg/mL	0.1 (0.1; 2.4)	0.1 (0.1; 0.8)
	SPD	pg/mL	23.9 (22.1; 29.6)	23.6 (17.8; 37.6)

PEx: concentration in 200 µL eluat from a half a filter membrane (PEx mean mass 120 ng). Concentration*0.2/120 ng will give the estimated concentration in ELF.

*%NS* % nonsquamous cells.

*p < 0.05,**p < 0.01,***p < 0.001. Median and IQR.

### PEx characterization and reproducibility between baseline visits

PEx were collected at screening and at two baseline visits prior to the respective LPS challenge (V1, V2, V5). We compared the baseline data of visit 2 and visit 5 to assess the reproducibility of PEx markers because subjects were unfamiliar with PEx sampling during their first visit. The numbers of PEx/L breaths and the PEx mass/L breaths showed a good correlation (r = 0.89, p = 0.001; r = 0.79, p = 0.006) between these visits, which were approximately five weeks apart. The total numbers of PEx/single breaths and the PEx mass/single breaths showed a similar result (r = 0.88, p = 0.001; r = 0.83, p = 0.003). The particle size distribution, expressed as the percentage of particles in each of the 8 size bins of the PExA particle counter, showed significant correlations (data not shown). Finally, we also detected good reproducibility for the concentration of SP-D in PEx between these baseline visits (r = 0.80, p = 0.01).

### PEx inflammatory response

PEx were collected twice after segmental and once following whole lung LPS challenge (Fig. 1). Five hours after the segmental challenge, we observed an increase in particle emission, and the PEx concentration and mass concentration were significantly higher at this time point than before (p = 0.0008) and 21 h after (p = 0.03) segmental challenge. The particle size distribution was not significantly affected. Only a trend (p = 0.08) for higher numbers of smaller particles (0.55–0.70 µm) was observed, paralleled by lower numbers in bins 4–8 (0.92–4.55 µm). No significant increase in PEx concentration or PEx mass was observed after the inhalation challenge, and no shift in PEx number distribution was observed.

**Figure 2:**
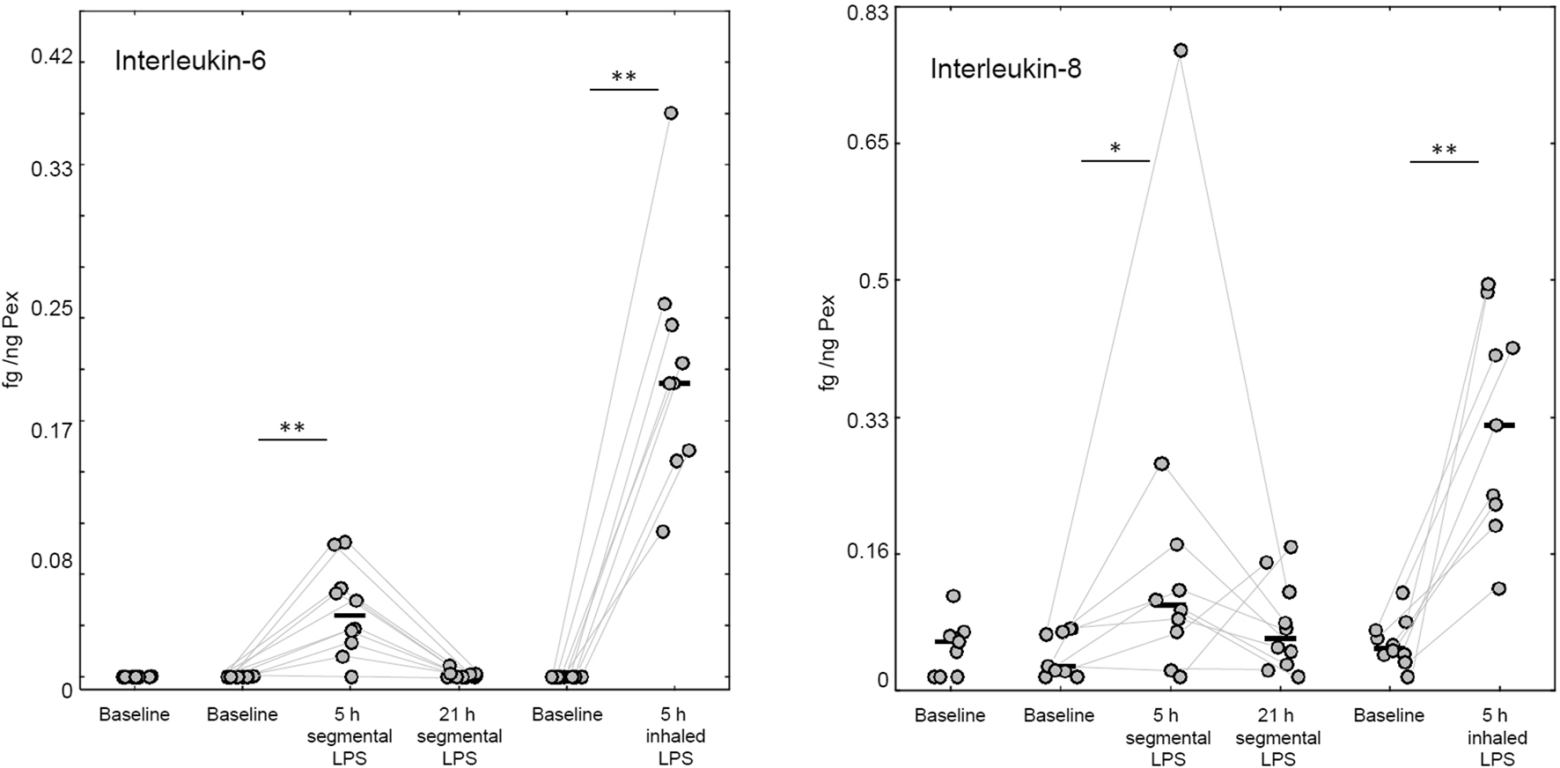
Individual and median concentrations of interleukin-6 and interleukin-8 per ng PEx. Two hundred microliters of elution buffer was used for a membrane with 120 ng of exhaled breath particles (PEx).*p < 0.05,**p < 0.01, Wilcoxon-matched pairs-test.

Despite very low concentrations of sample in the eluate from the collection membranes, we were able to detect SP-D and albumin in all samples using the MSD assay platform. Most MPO samples were below the detection limit of the available assay. This was also true for IL-6 and IL-8 with respect to most screening and baseline samples. However, a clear increase in the concentrations of IL-6 and IL-8 following segmental LPS challenge was detected. The response was even more pronounced after the inhalation challenge (Tables 1 and 2, Fig. 2).

## Discussion

In this study, we were able to demonstrate that it is possible to detect an LPS-induced inflammatory response in exhaled breath particles of healthy volunteer subjects. Using this controlled experimental approach and with the availability of novel sensitive analysis assays, we were able to detect an increase in IL-6 and IL-8 in very small amounts of undiluted ELF. Our data prove the concept that PEx analysis can be applied as a noninvasive monitoring tool in respiratory medicine.

### LPS challenge

In this study, we clearly showed the inflammatory response to both segmental and whole lung LPS challenge. As shown in previous studies, there was a clear increase in neutrophils and monocytes, which were detectable both in BAL fluid after segmental and in sputum after inhalation LPS challenge. The inflammatory response 24 h after segmental LPS challenge was also comparable with our previous study with respect to the five investigated biomarkers^2^.

In the segmental challenge, 200–400 ng LPS (depending on the subject’s weight) was applied to one lobe of the lung, and 2 µg was inhaled during the whole lung challenge. It is important to keep in mind that despite the massive inflammatory response in the one lobe, PEx are emitted and collected from the whole lung. Perhaps due to this, we observed a more pronounced increase in IL-6 and IL-8 following the inhalation challenge, which affects the entire lung and not only one lung segment. Interestingly, we observed a similar systemic response with respect to the increase in blood neutrophils following these different LPS challenges.

### PEx analysis

We aimed to collect 240 ng PEx at each time point. As shown in Table S1 (see Additional file 1), this target was missed several times at visit 1 (V1), when subjects were still unfamiliar with the procedure. In one subject, this target was missed repeatedly, which resulted in 2 missing values and overall the lowest detected concentrations of albumin and SP-D. A total of 120 ng was used for the analysis of proteins and extracted according to a protocol recommended by PExA AB. The strong correlation of SP-D concentrations between the baseline visits indicates that the extraction process was efficient and apparently resulted in a reproducible yield.

The analysis of PEx collected at the baseline visits showed that physical aerosol parameters such as particle number emissions and the particle size distribution are reproducible within individuals even over a period of more than 4 weeks. This has been demonstrated in more detail before^5,9^. The particle number emission increased five hours after the segmental LPS challenge, potentially related to the developing edema in the challenged segment.

Due to the limited amount of available PEx, we selected five biomarkers for our analysis. We chose IL-6 and IL-8, as these were known from previous studies^2,15^, to show a clear detectable increase in BAL fluid. Importantly, high-sensitive assays were available for both of these cytokines. In addition, we selected MPO as a further marker for neutrophilic inflammation^2,15^. Albumin was included as a control because it is abundant in ELF and was previously shown to be detectable in PEx^4^. SP-D is part of the pulmonary surfactant system and a dominant constituent of ELF but has previously been shown to not respond significantly to LPS challenge^2^. We did not analyse amylase in PEx due to the limited amount of material available and because it has been shown previously that PEx do not contain amalyse^16^. An extensive discussion on the origin of PEx and why a contamination of PEx with larger aerosols from the upper airways is extremely unlikely has been published by Ljungkvist et al.^14^.

To the best of our knowledge, this is the first time that the inflammatory cytokines IL-6 and IL-8 were detected in PEx. The increase is compatible with cytokine levels obtained from the analysis of BAL fluid, induced sputum and serum. As we did not have high and low responders to the LPS challenge in our study and with the need to limit the number of subjects to 10 in this complex study design, we were not able to find significant correlations between the LPS induced changes in BAL and PEx.

Based on the theoretical origin and generation of PEx, it can be assumed that PEx represent undiluted ELF^6^. Bronchoalveolar lavage also collects ELF from the peripheral lung. Based on Rennard et al.^17^, it can be estimated that with a standard lavage procedure, the volume of ELF recovered from a healthy human is approximately 1.0 ml in 100 ml recovered BAL fluid. Based on this, we calculated and compared the approximate concentrations in PEx and BAL. With the numerous assumptions required, with data from different studies and subjects, and with biomarkers being analyzed with different assays, this can only be a rather crude comparison. Nevertheless, as shown in Table S2 (see Additional file 1), the IL-6, IL-8 and SP-D data show a plausibly similar order of magnitude. The estimated concentrations of IL-6 and IL8 appeared to be lower in PEx at the relevant time point of 5 h after segmental LPS challenge. This would be expected, as PEx are sampled from the whole lung, which leads to dilution with ELF from unaffected lung areas. In contrast, the IL-6 and IL-8 response in BAL fluid originates from the challenged lobe only with no dilution from unaffected lung areas. Interestingly, the estimated SP-D concentration is quite comparable between PEx and BAL. As SP-D is not significantly affected by LPS, the concentration in the challenged lobe is not much different from that in the rest of the lung; therefore, comparable results would make sense. For albumin, the estimated concentrations are on the same order of magnitude but much more variable and sometimes higher in PEx than in BAL, which is difficult to explain with our current knowledge about PEx.

## Conclusion

In summary, this study has proven the concept that exhaled particles can be used for monitoring inflammatory changes in the lung, making PEx analysis an attractive noninvasive method for experimental lung research. With more sensitive biochemical analysis methods becoming available, the spectrum of potential analytes is constantly increasing. In addition, novel filter material and improved filter processing tools that were not available at the time of sample processing in our study are likely to further increase the yield of PEx for future applications. Due to the cost of the instrument, however, exhaled breath particle analysis will remain limited for use in general practice, but we do see a need for the noninvasive analysis of peripheral airway inflammation in clinical trials, especially for repeated evaluations where BAL analysis cannot be performed.

## Supplementary Information

Supplementary Information.

## Data Availability

The datasets generated and/or analyzed during the current study are not publicly available due to data protection reasons but are available from the corresponding author on request.

## Abbreviations

BAL: Bronchoalveolar lavage
ELF: Epithelial lining fluid
FEV_1_: Forced expiratory volume in 1 s
LPS: Lipopolysaccharide, bacterial endotoxin
IL6: Interleukin-6
IL8: Interleukin-8
IQR: Interquartile range
ISP: Induced sputum
LLOD: Lower limit of detection
MPO: Myeloperoxidase
MRI: Magnetic resonance imaging
MSD: Meso scale discovery
PEx: Particles in exhaled air
RIPA buffer: Radioimmunoprecipitation assay buffer
SD: Standard deviation
SP-D: Surfactant protein D
V: Study visit
