# Supplementary material for: Inflammatory cytokines can be monitored in exhaled breath particles following segmental and inhalation endotoxin challenge in healthy volunteers
Source: Sci Rep. 2022 Apr 4;12:5620. doi: 10.1038/s41598-022-09399-z (PMC8979977; doi:10.1038/s41598-022-09399-z)
Supplement: Supplementary file 1 — Supplementary Information. [file 41598_2022_9399_MOESM1_ESM.docx]

**Additional file 1**

BAL analysis

Bronchoscopy was performed according to international recommendations for fiberoptic bronchoscopy [1]. Subjects had to be in a fasting state (last meal at least 12 hours prior to bronchoscopy). During bronchoscopy and for two hours thereafter, all subjects were continuously monitored with a three-lead ECG, blood pressure measurements, and oximetry. Bronchoscopies were conducted under continuous oxygen supplementation. Two puffs of salbutamol (100 µg each) were applied 10 minutes prior to start of bronchoscopy and subjects were sedated with 0.05-0.1 mg/kg midazolam just prior to and during the procedure. Lidocaine (10%) spray and lidocaine solution (2%), up to 8.2 mg/kg were used to anesthetize upper and lower airways. During the first bronchoscopy, a baseline BAL from the left lower lobe was performed using 5 times 20 ml of 37°C sterile 0.9% saline. The aspirate was collected and stored on ice. During the second bronchoscopy, BAL from the challenged segments was collected in the manner described above.

Subjects spent at least four hours after each bronchoscopy in the institute for recovery and were examined by a physician before discharge. The BAL was filtered through a 100µm sieve, and centrifuged (300g 4°C). The supernatant was stored in aliquots at -80°C, the cells were counted in a hemocytometer. Cytospins were prepared using 20.000 cells and a differential cell count was performed after staining with Diffquick.

Induced sputum analysis

Sputum induction was performed as previously described [3]. Briefly, subjects inhaled 3 %, 4 % and 5 % hypertonic saline in 3 consecutive 10 min inhalation periods. After each period subjects were asked to produce sputum. Sputum plugs were selected, pooled and homogenized with Sputolysin® (Calbiochem, Darmstadt, Germany). After centrifugation, supernatants were collected and stored at −80 °C before the analysis of biomarkers. After filtration, cytospins were prepared and a differential cell count was performed by counting at least 400 non-squamous cells.

Inclusion criteria

Male and female healthy subjects, aged 18-65 years. Women will be considered for inclusion if they are:

Not pregnant, as confirmed by pregnancy test, and not nursing.

Of non-child bearing potential (i.e. physiologically incapable of becoming pregnant, including any female who is pre-menarchial or post-menopausal, with documented proof of hysterectomy or tubal ligation, or meets clinical criteria for menopause and has been amenorrhoeic for more than 1 year prior to the screening visit).

Of childbearing potential and using a highly effective method of contraception during the entire study.

• Normal lung function with FEV1 predicted ≥ 80%

• Nonsmokers with a history of less than 1 pack year having been nonsmokers for at least the last five years

• Able and willing to give written informed consent

Exclusion criteria

• Past or present disease, which as judged by the investigator, may affect the outcome of the study.

• Regular intake of any prescribed or over the counter medication. Exceptions include paracetamol for pain relief, oral contraceptive medication, hormonal replacement therapy, dietary and vitamin supplements

• Clinically relevant history of allergy as judged by the investigator

• Patient unable to undergo MRI

• Infections of the lower respiratory tract within 4 weeks before visit 1, visit 2, or visit 3.

• Any clinically relevant abnormal findings in physical examination, clinical chemistry, hematology, urinalysis, vital signs or ECG at Visit 1, which, in the opinion of the investigator, may either put the subject at risk because of participation in the study, or may influence the results of the study, or the subject’s ability to participate in the study

• Elevated IgE

• Intake of systemic or inhaled steroids in the previous 4 weeks before visit 1, visit 2, or visit 3

• History of drug or alcohol abuse

• Risk of non-compliance with study procedures

• Suspected inability to understand the protocol requirements, instructions and study-related restrictions, the nature, scope, and possible consequences of the study

**Table S1:** Individual PEx data

Table S2: Comparison of estimated marker ELF concentrations between BAL and PEx

The first 2 columns show that data of Ref [2] and the data of this study are comparable, despite different subjects being investigated and different assays being used. In the next 2 columns we first calculate the absolute amount of the respective markers. Based on Rennard et al. [3], we then estimate that about 0.6 ml ELF are contained in ~60mL BAL. As the markers are completely derived from ELF, we can compute the estimated concentration in ELF (shaded grey column). In this study no 6 h BAL data is available, therefore we calculate the same ELF concentration based on the data of Ref [2]. On the right hand side we calculated the ELF concentration based on the PEx data. The first column shows the data at 24 and 6 h as derived from Table 1 of this study. This data is then converted in the next column to the concentration in ELF by multiplying with 0.2 and division by 120 (200µL eluate correspond to a mean of 120 ng PEx). We next convert ng ELF into pL ELF based on the assumption that ELF has a density of 1g/mL. In the next column we compute the concentration in 1 mL of ELF by multiplication with 1E9. And finally the grey shaded column shows the concentration that can be compared to the concentration estimated from BAL fluid. The closest agreement appears to be for SPD, which is explained in the discussion. The rest of the data is at least in the same order of magnitude.

**1** Workshop summary and guidelines: investigative use of bronchoscopy, lavage, and bronchial biopsies in asthma and other airway diseases. *J Allergy Clin Immunol.* 1991;(88(5)): 808–814.

**2** Holz O, Tan L, Schaumann F, Müller M, Scholl D, Hidi R, McLeod A, Krug N, Hohlfeld JM. Inter- and intrasubject variability of the inflammatory response to segmental endotoxin challenge in healthy volunteers. *Pulmonary pharmacology & therapeutics* 2015; 35: 50–59.

**3** Rennard SI, Basset G, Lecossier D, O'Donnell KM, Pinkston P, Martin PG, Crystal RG. Estimation of volume of epithelial lining fluid recovered by lavage using urea as marker of dilution. *Journal of applied physiology (Bethesda, Md. : 1985)* 1986; 60: 532–538.
